# Phytochemical Profiling, Antioxidant Capacity, and the Antidiabetic Potential of *Atriplex halimus* Extracts: UHPLC – MS / MS Analysis, In Vitro Enzyme Inhibition Assays, and Molecular Docking Studies

**DOI:** 10.1002/fsn3.70745

**Published:** 2025-08-02

**Authors:** Mohammed Roubi, Nour Elhouda Daoudi, Mohammed Dalli, Salah‐eddine Azizi, Youness Mahdi, Ramzi A. Mothana, Hanan M. Al‐Yousef, Mohammed F. Hawwal, Fahd Kandsi, Raffaele Conte, Nadia Gseyra

**Affiliations:** ^1^ Laboratory of Bioresources, Biotechnology, Ethnopharmacology and Health Mohammed First University Oujda Morocco; ^2^ Higher Institute of Nursing Professions and Health Techniques Oujda Morocco; ^3^ Department of Pharmacognosy, College of Pharmacy King Saud University Riyadh Saudi Arabia; ^4^ Research Institute on Terrestrial Ecosystems (IRET)‐CNR Naples Italy

**Keywords:** α‐amylase, antidiabetic, *Atriplex halimus*, hemoglobin glycation

## Abstract

*Atriplex halimus* (L.) is known to be characterized by numerous pharmacological prospects comprising antioxidant, anticancer, antibacterial, and antidiabetic properties. The core objective of the present study is to harness the chemical composition of aqueous extract (EA), hydroethanolic extract (EHA), and ethanolic extract (EE), followed by the investigation of the antidiabetic potential of *A. halimus* extracts on two important targets, porcine proteins (α‐amylase and hemoglobin). A molecular docking study was adopted to computationally identify the bioactive compounds responsible for the observed antidiabetic effect. Phytochemical profiling using UHPLC–MS/MS identified 21 compounds across the three extracts, with trans‐cinnamic acid being the most abundant in all. The extracts exhibited significant inhibitory effects on both α‐amylase and hemoglobin glycation, with IC_50_ values of 1.89, 3.33, and 2.07 mg/mL (for EA, EHA, and EE, respectively) against α‐amylase and 0.29, 0.31, and 0.41 mg/mL against glycation, demonstrating a significant inhibitory impact of *A. halimus* on both target proteins. With regard to the in silico computational study, it has been demonstrated that both catechin and epigallocatechin emerge as the most active compounds, displaying high binding activity compared to acarbose, a standard antidiabetic drug. These findings highlight the rich phytochemical profile and strong antidiabetic potential of *A. halimus* extracts, supporting their development as natural therapeutic agents for diabetes management.

## Graphical abstract

*Atriplex halimus* extracts exhibited significant antidiabetic activity, notably inhibiting α‐amylase and hemoglobin glycation. *UHPLC‐MS/MS* analysis identified 21 phytochemicals, with trans‐cinnamic acid being the most abundant. Molecular docking revealed catechin and epigallocatechin as key bioactive compounds with strong binding affinities, surpassing the standard drug acarbose.

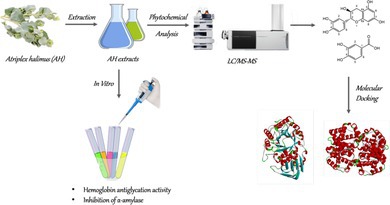

## 1 Introduction

According to the World Health Organization (WHO), it is anticipated that the global diabetic population will double within the next 30 years. The Middle East currently bears the highest prevalence of diabetes, with 15.2 million individuals affected in the Year 2000. Prognostics indicate that by 2030, the number of people with diabetes in the Middle East will nearly triple, reaching 42.6 million, signifying a substantial increase from the baseline of 15.2 million in 2000 (Dalli et al. 2021; Kadan et al. 2013). In recent decades, the increasing prevalence of diabetes has had a growing impact on human health and development, leading to a greater burden on families and society worldwide (Zeng et al. 2025). Diabetes is a chronic condition marked by elevated blood glucose levels and abnormal protein and fat metabolism. This occurs either due to insufficient insulin production by the pancreas or the cells' inability to effectively utilize the produced insulin, leading to elevated blood sugar levels (Dalli et al. 2022; Roglic 2016). There are numerous types of DM, including type 1 and type 2. Two classifications for type 1 diabetes are suggested by the American Diabetes Association committee: type 1A for immune‐induced diabetes with loss of the pancreatic islet β‐cells and type 1B for nonimmune‐mediated diabetes with severe insulin deficiency (Devendra et al. 2004). In type 2 diabetes, this primarily appears as the body becoming resistant to insulin, the hormone necessary for processing the sugar glucose (Ahmad et al. 2022; Wei et al. 2024). Over the past two decades, the prevalence of type 2 diabetes has doubled, emerging as a significant concern with almost 90% of the worldwide diabetes cases (Ahmad et al. 2022). The oxidative stress, on the other hand, plays a significant role in the complications of diabetes; the lipid peroxidation, for example, can play a crucial role in the development of atherosclerosis by a cascade of events such as the production of reactive carbonyl species, recruitment and activation of macrophages, cellular proliferation, and the modification of vascular proteins by lipoxidation end‐products. This series of events contributes collectively to the development of atherosclerosis (Baynes and Thorpe 2000). *Atriplex halimus L*. (AH) is one of the herbs used to treat diabetes (Day 1998; Roubi, Dalli, et al. 2024). AH has long been a well‐liked home remedy and was used especially by the Arab community to treat different diseases (Chaachouay et al. 2021) such as intestinal worms (Ghourri et al. 2012), control gall bladder excretions (Lakhdari et al. 2016), relieve stomach pain (Le Houérou 1992), and treat chest conditions (Roubi et al. 2023). Numerous conducted researches in the field have demonstrated great richness of AH with a multitude of bioactive compounds, such as phenolic acids, flavonoids known for their different biological potential, such as quercetin, which showed a potential therapy for diabetic encephalopathy (Azizi et al. 2024; Bouaziz et al. 2021; Cheng et al. 2024; Dalli et al. 2024) and also alkaloids (Benhammou et al. 2009). Regarding the pharmacological properties, this plant has been shown to be endowed with antioxidant (Chaouche et al. 2021), anticancer, antibacterial (Bouaziz et al. 2021), and antidiabetic activities (Bounouar et al. 2022). The main objective of this study was to investigate the phytochemical composition of *A. halimus* aqueous extract (EA), hydroethanolic extract (EHA), and ethanolic extract (EE) utilizing UHPLC–MS/MS. Additionally, the study aimed to explore the in vitro antioxidant activity of the EE extract and antidiabetic potential of EE, EA, and EHA by assessing their ability to inhibit porcine pancreatic α‐amylase and prevent human hemoglobin glycation. Molecular docking analysis was employed to elucidate the mechanism of action and identify the bioactive components responsible for the reported antidiabetic benefits.

## 2 Materials and Methods

### 2.1 Plant Identification

*Atriplex halimus* (L.) was collected in accordance with the guidelines and regulations established by the Plant Varieties Protection. Professor El Achouri, as a botanist, identified it, and the specimen was delivered to the faculty of science, Oujda's herbarium with the identification code HUMPOM786.

### 2.2 Extraction Procedure

The aerial parts of *A. halimus* were dried in a shaded room. Subsequently, the dried plant material was finely powdered using a milling machine (model SM‐450, China). Following this, 100 g of the dried plant material was combined with a 70% ethanol solution for approximately 48 h in amber flasks. The resulting liquid, after filtration, underwent vacuum drying using a rotary evaporator, yielding the hydroethanolic crude extract (EHA).

The same procedure was employed to prepare the aqueous extract (EA), substituting distilled water for the ethanol solution. Similarly, the ethanolic extract (EE) was prepared by soaking 100 g of the powdered material in absolute ethanol for 48 h. All three extracts were filtered, concentrated as necessary, and stored at 4°C for future analysis.

### 2.3 Qualitative and Semi‐Quantitative Analysis of *A. halimus* Using UHPLC‐MS/MS

Sample aliquots of EA, EHA, and EE, each weighing 80 mg, were subjected to the following extraction procedure: Each aliquot was treated with 1 mL of ethanol, then the mixture was agitated using a vortex mixer and subsequently sonicated for 60 min at 45°C. Qualitative chromatographic analyses were performed using a Shimadzu Ultra‐High‐Performance Liquid Chromatograph (Nexera XR LC 40, Kyoto, Japan) coupled with an MS/MS detector (LCMS 8060, Shimadzu Italy, Milan, Italy). The detector's electrospray ionization functionality was regulated by the Lab Solution software (version 5.6, Kyoto, Japan), enabling the swift transition within a singular LC cycle from a low‐energy scan at 4 V (full scan MS) to a ramped high‐energy scan ranging from 10 to 60 V. The instrumental parameters were defined as follows: 2.9 L/min for nebulizing gas, 10 L/min for heating gas, 250°C for the DL, 300°C for the interface, 400°C for the heat‐block, and 10 L/min for drying gas. Chromatographic separation was obtained using a mixture of acetonitrile and water, with an inclusion of 0.01% formic acid, maintaining a ratio of 5:95 (v/v) as mobile phase and a Kinetex 2.6 μm Polar C18 LC column (Phenomenex Inc., USA) as stationary phase. Instrumental settings were tailored for a SIM experiment in the negative ion mode (López‐Fernández et al. 2020; Mechchate et al. 2021). Compounds were identified by juxtaposing the characteristic molecular weights and retention times with our proprietary molecular database. A molecule was deemed positively identified if its area under the curve surpassed that of the control blank. Time of flight was used to distinguish between extremely similar structures because the equipment was programmed to measure the molecular weight in the third quadrupole.

### 2.4 Antioxidant Activity

#### 2.4.1 Free Radical Scavenging Test

The radical scavenging capacity of the *Atriplex halimus* ethanolic extract (EE) was evaluated using the DPPH assay as described in (Roubi, Azizi, et al. 2024) with some modifications. 4 mg of DPPH and 100 mL of methanol were mixed to create the stock solution, and we also prepared a serial dilution of EE that ranged from 0.25 to 2 mg/mL. We mixed 1.5 mL from the stock solution with 0.5 mL of each concentration and then incubated the mixture in the dark at room temperature for 30 min. We measured the absorbance at 517 nm once the incubation time was over, and we compared it to the blank. Ascorbic acid was used as a positive control.

The inhibition percentage was calculated according to the formula:

$$\%\text{of inhibition}=\left[\left(\frac{B-A}{B}\right)\right]\times 100$$

*A*: Absorbance of the sample at 517 nm. *B*: Absorbance of the blank.

#### 2.4.2 Determination of β‐Carotene Bleaching

β‐Carotene's antibleaching activity was assessed using the methodology described in (Roubi et al. 2023). The following were dissolved in 10 mL of chloroform: 20 mg of linoleic acid, 2 mg of β‐carotene, and 200 mg of Tween 80. The chloroform was removed from the mixture using a rotary evaporator at 40°C. Subsequently, 100 mL of distilled water was vigorously added. Subsequently, the chloroform was eliminated from the mixture using a rotary evaporator that operated at 40°C. 0.5 mL of the various concentrations of EE was combined with 2.5 mL of the β‐carotene solution. The absorbance was promptly measured at 470 nm using a spectrophotometer after the extract was added (t0). Subsequently, the samples were incubated at 50°C for 2 h, and the absorbance was measured at 470 nm (t1). Butylated hydroxyanisole (BHA) was used as a positive control.

Utilizing the formula, the residual color % was calculated:

$$\text{Bleaching assay}\;\left(\%\right)=\left[\left(\frac{\mathrm{OD}\;{\mathrm{at}}_{\text{to}}-\mathrm{OD}\;{\mathrm{at}}_{t1}}{\mathrm{OD}\;{\mathrm{at}}_{t0}}\right)\right]\times 100$$

#### 2.4.3 Total Antioxidant Assay

The phosphor‐molybdenum technique, as described in (Chaudhary et al. 2015), was employed to assess the overall antioxidant activity of *Atriplex halimus* ethanolic extract. The reagent was prepared using 0.6 M sulfuric acid, 28 mM sodium phosphate, and 4 mM ammonium molybdate. 0.3 mL of this reagent was mixed with 0.1 mL of EE and then incubated for 90 min at 60°C. Once the incubation time was over and after cooling at room temperature, we measured the absorbance at 695 nm. The standard curve was created using ascorbic acid, and the outcomes were expressed as ascorbic acid equivalents (Prieto et al. 1999). Every experiment was run three times.

### 2.5 Antidiabetic Activity

#### 2.5.1 Inhibition of α‐Amylase In Vitro

By adopting the protocol published by (Daoudi et al. 2020), the inhibitory potential of *Atriplex halimus* extracts on α‐amylase enzyme was evaluated. 200 μL of EA, EHA, EE, and/or acarbose used as positive control were added to 200 μL of PBS (pH = 6.9) and 200 μL of pancreatic α‐amylase enzyme in order to prepare the reaction mixture. This solution was then pre‐incubated for a duration of 10 min at 37°C. Afterward, 200 μL of a 1% starch solution was added to each tube, followed by an incubation period at 37°C lasting 20 min. The enzymatic reaction was stopped by adding 600 μL of DNSA.

The various tubes were incubated at 100°C for about 8 min, and then quickly cooled in an ice bath. At the end, the tubes containing the various test extracts (EHA, EA, EE, and acarbose) were diluted by adding 1 mL of distilled water. We then measured the absorbance of the mixture at 540 nm. The inhibition percentage of α‐amylase enzyme was calculated according to the formula:

$$\text{Inhibition percentage}\;\left(\%\right)=\frac{\left(\text{ODcontrol}-\text{ODtest}\right)}{\text{ODcontrol}}\;x\;100$$

#### 2.5.2 Hemoglobin Antiglycation Activity In Vitro

We used the procedure outlined by (Dalli et al. 2022) to assess the hemoglobin antiglycation potential of *A. halimus* extracts. An aliquot of 5 μL of gentamicin, 25 μL of each extract (EHA, EA, and EE), or gallic acid at various concentrations, followed by PBS (pH 7.4), was added to 1 mL of the 5% hemoglobin solution. Subsequently, an aliquot of glucose (1 mL) (4 mg/mL) was added to start the reaction. Then, the reaction mixture was kept in the dark, in a room‐temperature environment for 72 h. The absorbance of the combination was determined at 443 nm. The inhibition % was calculated using the formula below:

$$\text{Inhibition percentage}\;\left(\%\right)=\frac{\left({\mathrm{OD}}_{\text{control}}-{\mathrm{OD}}_{\text{Blank}}\right)-\left({\mathrm{OD}}_{\text{Sample}}-{\mathrm{OD}}_{\text{sample blank}}\right)}{{\mathrm{OD}}_{\text{Control}}-{\mathrm{OD}}_{\text{Control Blank}}}\;x\;100$$

### 2.6 Molecular Docking

For a comprehensive understanding of the pharmacological effects observed in vitro, the molecular docking approach emerges as the most suitable technique. This method facilitates the study of potential interactions between the bioactive compounds identified in each extract and the active pocket of each target protein implicated in diabetes (Pires et al. 2015). The three‐dimensional (3D) structures of the primary chemical compounds found in EA, EHA, and EE were retrieved from PubChem (accessed on September 5, 2023) for utilization in the docking analysis. The molecular structures of the principal bioactive compounds were procured from the PubChem database in the form of “3D sd” files. Subsequent conversion to the “pd” format was executed utilizing PyMol, a sophisticated tool designed for molecular visualization and analysis. Porcine pancreatic alpha‐amylase (1OSE) and human hemoglobin protein (2D60) were chosen as the study's target proteins, whose crystallographic forms were retrieved from the Protein Data Bank website (accessed on September 5, 2023) using their unique PDB IDs. The protein structures were viewed using the Discovery Studio 4.1 software. The proteins were produced utilizing a three‐step conventional process prior to the molecular docking investigation. In addition, water molecules (H_2_O) were removed from the protein structures, and polar hydrogen bonds and Kollmann charges were added in order to improve the model's accuracy and take electrostatic interactions into consideration during the docking process.

The AutoDock Vina v1.5.6 software was used to carry out the molecular docking in silico experiment. The protein and ligand data were transformed into three‐dimensional PDBQT files using MGL methods. Then, grid maps were created using the AutoGrid tool, which is a crucial part of the AutoDock software package. Throughout the molecular docking process, these maps show the interaction energy between the ligands and the target macromolecule (Trott and Olson 2010).

### 2.7 Pharmacokinetic‐ADMET Analysis

Pharmacokinetic‐ADMET analysis was performed on the major compounds in the *Atriplex halimus* extracts; the PubChem website was used to obtain the isomeric smiles of these compounds, while the pkCSM—pharmacokinetics website was used to determine the ADMET study (Roubi, Dalli, et al. 2024).

## 3 Results and Discussion

### 3.1 UHPLC–MS/MS Results of *A. halimus* Extract's

**FIGURE 1:**
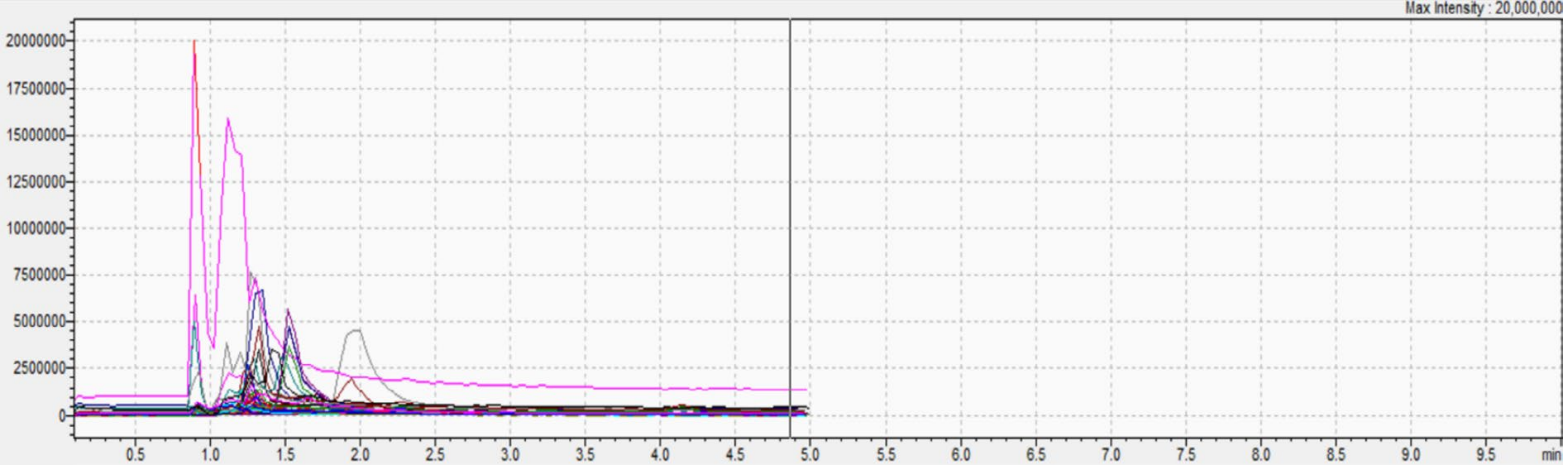
UHPLC–MS/MS spectrum of each compound of EA.

**FIGURE 2:**
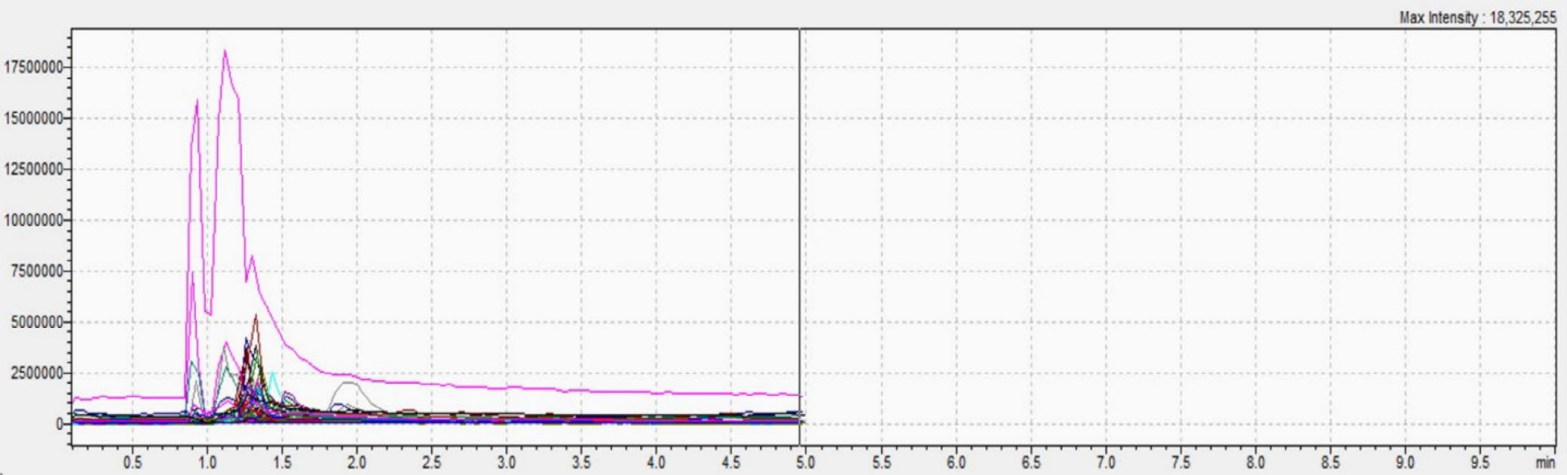
UHPLC–MS/MS spectrum of each compound of EHA.

**FIGURE 3:**
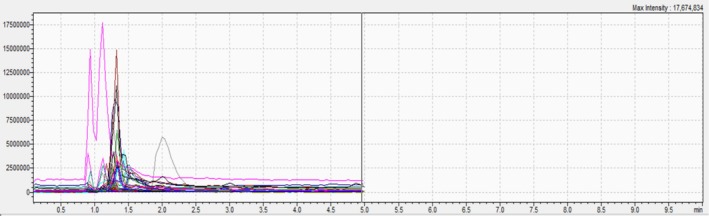
UHPLC–MS/MS spectrum of each compound of EE.

The results of UHPLC–MS/MS are depicted in (Table 1 and Figures 1, 2, and 3). The chemical analysis allowed the identification of 21 compounds with different levels of abundance. Hence, trans‐cinnamic acid was the major compound in all three extracts. On the other hand, EHA and EA demonstrated large diversity, with the bulk of the compounds being present in moderate to high abundance. However, trans‐ferulic acid and luteolin were noticeably lacking in these two substances. The identified compounds were divided into three main groups:

**TABLE 1:** Composition of *A. halimus* EA, EHA, and EE using UHPLC–MS/MS.

	Molecular formula	Molecular weight	[M‐H]‐	Quantity in μg/g of extract		
				EA	EHA	EE
Trans‐cinnamic acid	C_9_H_8_O_2_	148.16	147	73,73	84,02	48,88
Epigallocatechin	C_15_H_14_O_7_	306.27	305	22,98	12,49	35,10
Oleocanthal	C_17_H_20_O_5_	304.34	303.2	16,63	8,97	13,97
Gentisic acid	C_7_H_6_O_4_	154.12	153	13,01	5,23	9,18
Catechin	C_15_H_14_O_6_	290.27	289	15,43	8,19	8,44
Protocathecoic acid	C_7_H_6_O_4_	154.12	153	14,43	5,04	9,02
p‐Coumaric acid	C_9_H_8_O_3_	164.16	162.9	12,14	8,38	5,02
Hydroxytyrosol	C_8_H_10_O_3_	154.16	153.05	17,46	6,60	11,10
Trans ferulic acid	C_10_H_10_O_4_	194.18	193	0,65	0	0
Hesperetin	C_16_H_14_O_6_	302.28	301.3	2,21	3,34	1,48
Rosmarinic acid	C_18_H_16_O_8_	360.3	359	1,51	3,01	3,23
Ursolic acid	C_30_H_48_O_3_	456.7	455	0,81	0,75	1,52
Apigenin	C_15_H_10_O_5_	270.24	269	0,55	0,28	0,52
Luteolin	C_15_H_10_O_6_	286.24	284.9	1,51	0,82	0
Kaempferol‐3‐O‐glucose	C_21_H_20_O_11_	448.4	447	0	3,40	4,61
Quercetin‐3‐O‐hexose deoxyhexose	C_27_H_30_O_15_	594.5	593	0	3,12	4,71
Isorhamnetin‐3‐O rutinoside	C_28_H_32_O_16_	624.5	623.1	0	4,79	5,44
Kaempferol‐3‐O‐pentose	C_20_H_18_O_10_	418.3	417.1	4,95	4,46	3,21
Tyrosol	C_8_H_10_O_2_	138.16	137	10,10	3,94	7,35
Syringic acid	C_9_H_10_O_5_	198.17	197	0,56	0,48	0,88
Rutin	C_27_H_30_O_16_	610.5	609	0	2,66	4,39

Group 1: Common compounds (present in all three extracts: EA, EHA, and EE): p‐coumaric acid, oleocanthal, hydroxytyrosol, hesperetin, rosmarinic acid, ursolic acid, apigenin, tyrosol, protocatechuic acid, syringic acid, kaempferol‐3‐o‐pentose, gentisic acid, trans‐cinnamic acid, catechin, epigallocatechin.

Group 2: Compounds (present in only two of the extracts): Luteolin (present in EA and EHA) rutin; quercetin‐3‐o‐hexose deoxyhexose; isorhamnetin‐3‐o‐rutinoside; kaempferol‐3‐o‐glucose (present in EHA and EE).

Group 3: Unique compounds (present in only one of the extracts): EA: trans ferulic acid.

P‐Coumaric acid and Trans‐Cinnamic acid are the major phenolic acids that were detected in *Atriplex halimus* extracts (Bouaziz et al. 2021), which correlate perfectly with our results. In our research, the compounds that we discovered can be categorized into six distinct classes of molecules, each of which is comprehensively described in Table 2.

**TABLE 2:** *A. halimus* extracts compounds classification.

Class	Compounds
Phenolic acids	Trans‐cinnamic acid, trans‐ferulic acid, protocatechuic acid, gentisic acid, syringic acid, rosmarinic acid, p‐coumaric acid
Aliphatic alcohols	Hydroxytyrosol, tyrosol
Triterpene	Ursolic acid
Flavonoids	Quercetin‐3‐O‐hexose deoxyhexose, hesperetin, apigenin, luteolin, kaempferol‐3‐O‐pentose, kaempferol‐3‐O‐glucose, catechin, epigallocatechin, isorhamnetin‐3‐O rutinoside, rutin
Phenolic aldehydes	Oleocanthal

### 3.2 Antioxidant Activity

In our previous study (Roubi et al. 2023), we evaluated the antioxidant potential of both EA and EHA. The hydroethanolic extract (EHA) showed the best antioxidant potential in all the assays (IC_50_^DPPH^ = 0,59 ± 0,12 mg/mL; IC_50_^BC^ = 2,21 ± 0,22 mg/mL and with a total antioxidant capacity equal to 223.2 ± 2.45 μg AA/mg Ex). In this work, we evaluated the antioxidant potential of the ethanolic extract using the same methods used in the previous study; the ethanolic extract (EE) showed an interesting antioxidant potential with an IC_50_ in the DPPH assay equal to 0.75 ± 0.18 mg/mL. EE also showed potential in the prevention of the peroxidation of beta‐carotene with IC_50_ = 3.29 ± 0.26 mg/mL. The total antioxidant capacity was also performed, and EE showed an interesting result of 182.4 ± 3.12 μg AA eq/mg extract, which reinforces the findings from the other antioxidant assays. The results suggest that *Atriplex halimus* ethanolic extract has the ability to scavenge free radicals and can also offer protection against lipid peroxidation. Both of the studies showed that *Atriplex halimus* has great potential as a plant with antioxidant activity; EHA showed the best results, but still, EE and EA also demonstrated potential as agents against oxidative damage.

### 3.3 Antidiabetic Properties

#### 3.3.1 α‐Amylase Inhibition In Vitro

**FIGURE 4:**
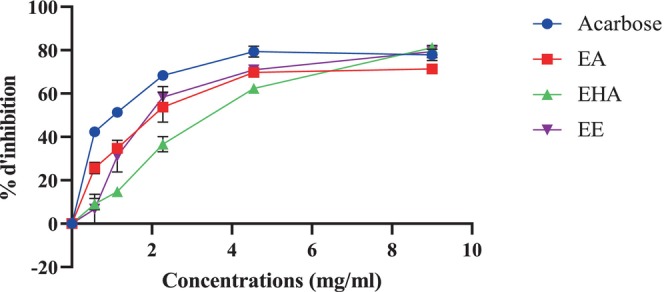
*Atriplex halimus* extracts and acarbose against pancreatic α‐amylase.

**FIGURE 5:**
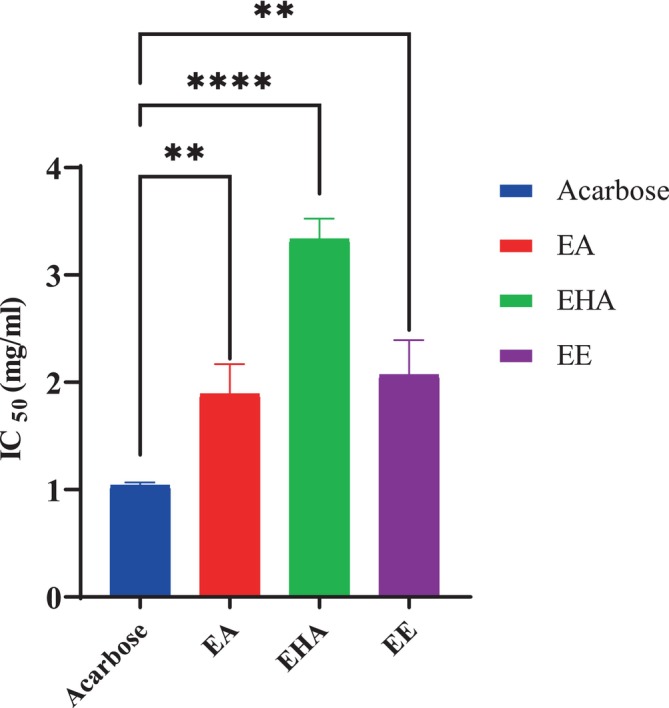
IC_50_ of *Atriplex halimus* extracts and acarbose. Each value is reported as mean ± SEM (*n* = 3). *p* < 0,01, ****p < 0,0001.

The porcine pancreatic α‐amylase was shown to be significantly inhibited by all extracts, EA, EHA, and EE, in a concentration‐dependent manner (Figure 4). At a high concentration of 9 mg/mL, EA, EHA, and EE showed inhibition percentages of 71.73% ± 1.56%, 81.12% ± 0.96%, and 79.39% ± 2.89%, respectively. Notably, acarbose demonstrated a slightly lower inhibition percentage with a value of 77.8% ± 2.65% at the same concentration. The IC_50_ values recorded for EA, EHA, and EE were 1.89 ± 0.27, 3.33 ± 0.19, and 2.069 ± 0.32 mg/mL, respectively (Figure 5). The best IC_50_ value was found to be 1.03 ± 0.02 mg/mL for acarbose, which was utilized as a positive control. The findings of the α‐amylase inhibitory activity indicate that EHA exhibits significant inhibition action at increasing concentrations, whereas EA demonstrates the most favorable IC_50_ value. The aqueous and ethyl acetate extracts of *Gracilaria bursa‐pastoris* showed an IC_50_ = 0.85 ± 0.01 and 1.86 ± 0.06 mg/mL, respectively, which was slightly close to the results obtained in our study (Ouahabi et al. 2023). The statistical analysis revealed a highly significant difference between the IC_50_ of acarbose, used as a positive control, and the IC_50_ of EHA (*p* < 0,0001) and EA and EE (*p* < 0.01).

#### 3.3.2 Hemoglobin Antiglycation Activity In Vitro

**FIGURE 6:**
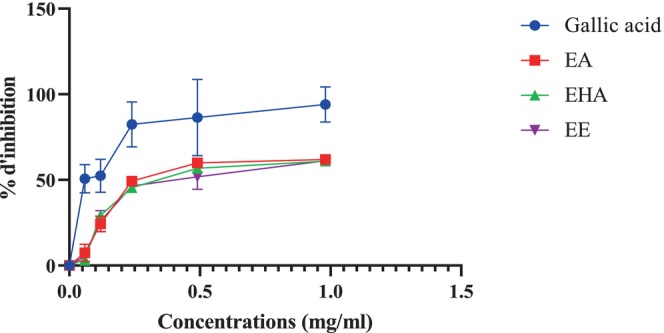
*Atriplex halimus* extracts and gallic acid hemoglobin antiglycation activity.

**FIGURE 7:**
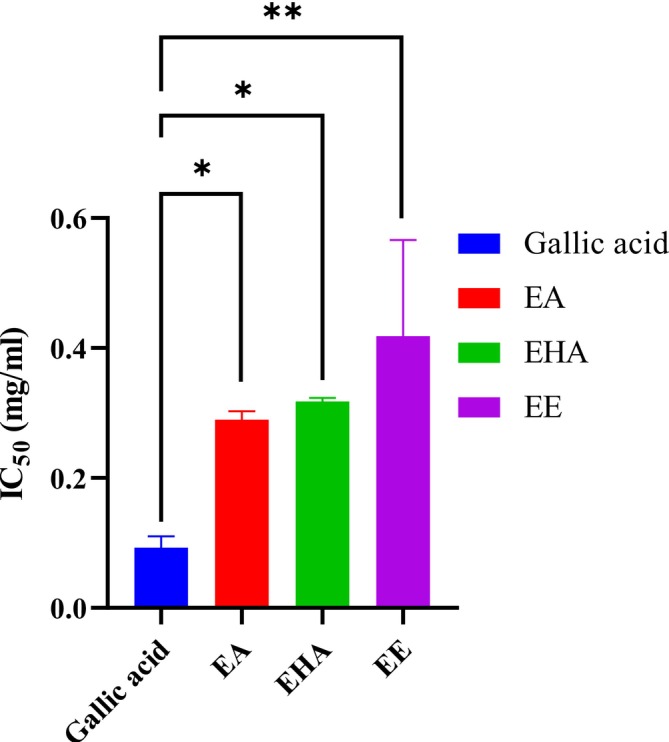
IC_50_ of *Atriplex halimus* extracts and gallic acid. Each value is reported as mean ± SEM (*n* = 3). *p < 01, **p < 0.0.1.

The hemoglobin antiglycation technique was used to test the in vitro antiglycation effect of the *A. halimus* extracts. According to the results, hemoglobin glycation was inhibited in a dose‐dependent manner (Figure 6). EA, EHA, and EE showed an inhibition of 61.98% ± 0.77%, 60.91% ± 0.91%, and 61.21% ± 0.82%, respectively, while gallic acid showed an inhibition percentage of roughly 94.08% ± 1.24% at a concentration of 0.98 mg/mL. The IC_50_ value recorded for EA was 0.29 ± 0.013 mg/mL, 0.31 ± 0.005 mg/mL for EHA, 0.41 ± 0.14 mg/mL for EE, and 0.09 ± 0,017 mg/mL for gallic acid (Figure 7). Our extracts showed a better activity compared to *Solanum elaeagnifolium* leaf extracts, which showed an IC_50_ = 3.990 ± 0.236 mg/mL (Bouslamti et al. 2023). The statistical analysis performed showed the existence of a significant difference between the IC_50_ of the gallic acid used as a positive control and the IC_50_ of EE (*p* < 0,01) and EA, EHA (*p* < 0.01).

### 3.4 Molecular Docking

#### 3.4.1 Porcine Pancreatic Alpha‐Amylase (1OSE)

Understanding the binding mode and likely binding position of a specific drug within protein scaffolds is crucial for assessing and comprehending the molecule's effectiveness as a therapeutic agent (Paul et al. 2014). The α‐amylase enzyme is known to catalyze the conversion of starch to simple sugar; its inhibition plays a key role in managing type 2 diabetes (Keerthana et al. 2013). Conventional inhibitors of α‐amylase, such as acarbose, miglitol, and voglibose (Kaur et al. 2021), have been found to cause significant side effects, including bloating (Aoki et al. 2010), obesity, and abdominal pain (Dabhi et al. 2013). The assessment of the molecular docking study could provide significant results of new candidates among the different bioactive compounds identified in *A. halimus* using UHPLC–MS/MS. Acarbose, used as a native ligand, showed a high binding energy of −8.0 kcal/mol. Meanwhile, catechin and epigallocatechin showed the highest binding energy with −9.3 and −8.3 kcal/mol, respectively, which was higher in comparison with the control (Table 3).

**TABLE 3:** *Atriplex halimus* ethanolic extract antioxidant assay results.

Extract/reference	DPPH^mg/mL^	β‐Carotene^mg/mL^	TAC^μg AA eq/mg extract^
EE	0.75 ± 0.18	3.29 ± 0.26	182.4 ± 3.12
Ascorbic acid (AA)	0.025 ± 0.05	—	—
Butylated hydroxyanisole	—	0.084 ± 0.02	—

**FIGURE 8:**
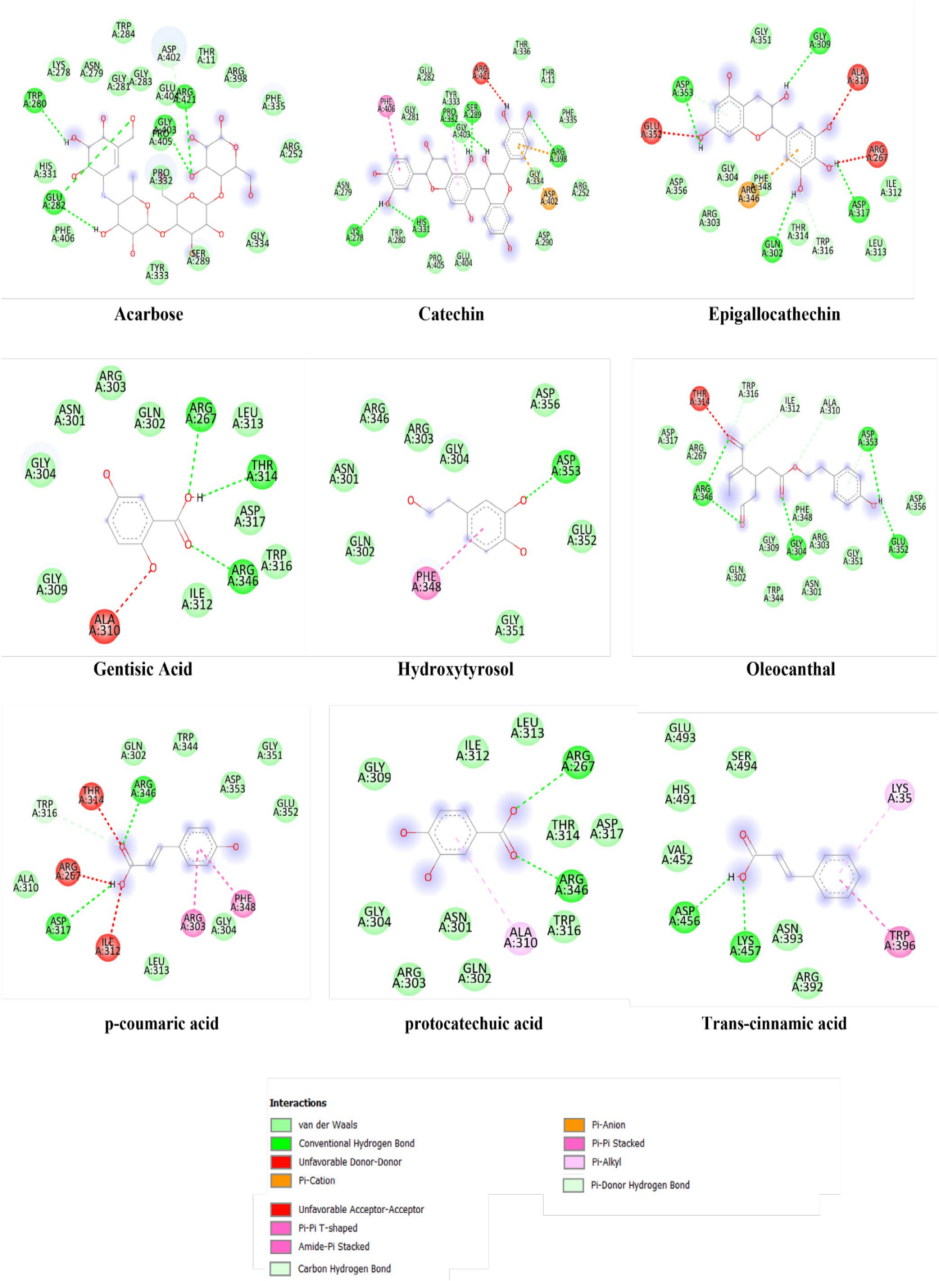
Ligands interactions with porcine pancreatic α‐amylase (1OSE).

This result indicates that our extracts are rich in substances like catechin, which was prevalent in the EA, EHA, and epigallocatechin, which was plentiful in both the EE and the EA and which, individually or synergistically, reduce α‐amylase activity. Figure 8 shows the chemical bonding manner of the complexes generated between the investigated chemicals and the binding pocket residues of α‐amylase. While the acarbose links in the active site of 1OSE with interaction like: TRP280, HIS331, GLU282, PHE406, TYR333, SER289, GLY334, ARG252, PHE335, ARG398, THR11, ASP402, ARG421, GLU404, GLY403, PRO405, PRO332, GLY283, GLY281, TRP284, ASN279, LYS278, catechin, and epigallocatechin engage with interaction like: ASN279, LYS278, TRP280, HIS331, PRO405, GLU404, ASP290, ARG252, ASP402, GLY334, ARG398, PHE335, THR11, THR336, SER289, GLY403, PRO332, TYR333, GLU282, GLY281, PHE406, and ASP353, GLY351, GLY309, ILE312, ASP317, LEU313, TRP316, THR314, GLN302, PHE348, ARG346, GLY304, ARG303, ASP356, respectively (Figure 8).

#### 3.4.2 Human Hemoglobin Protein (2D60)

**FIGURE 9:**
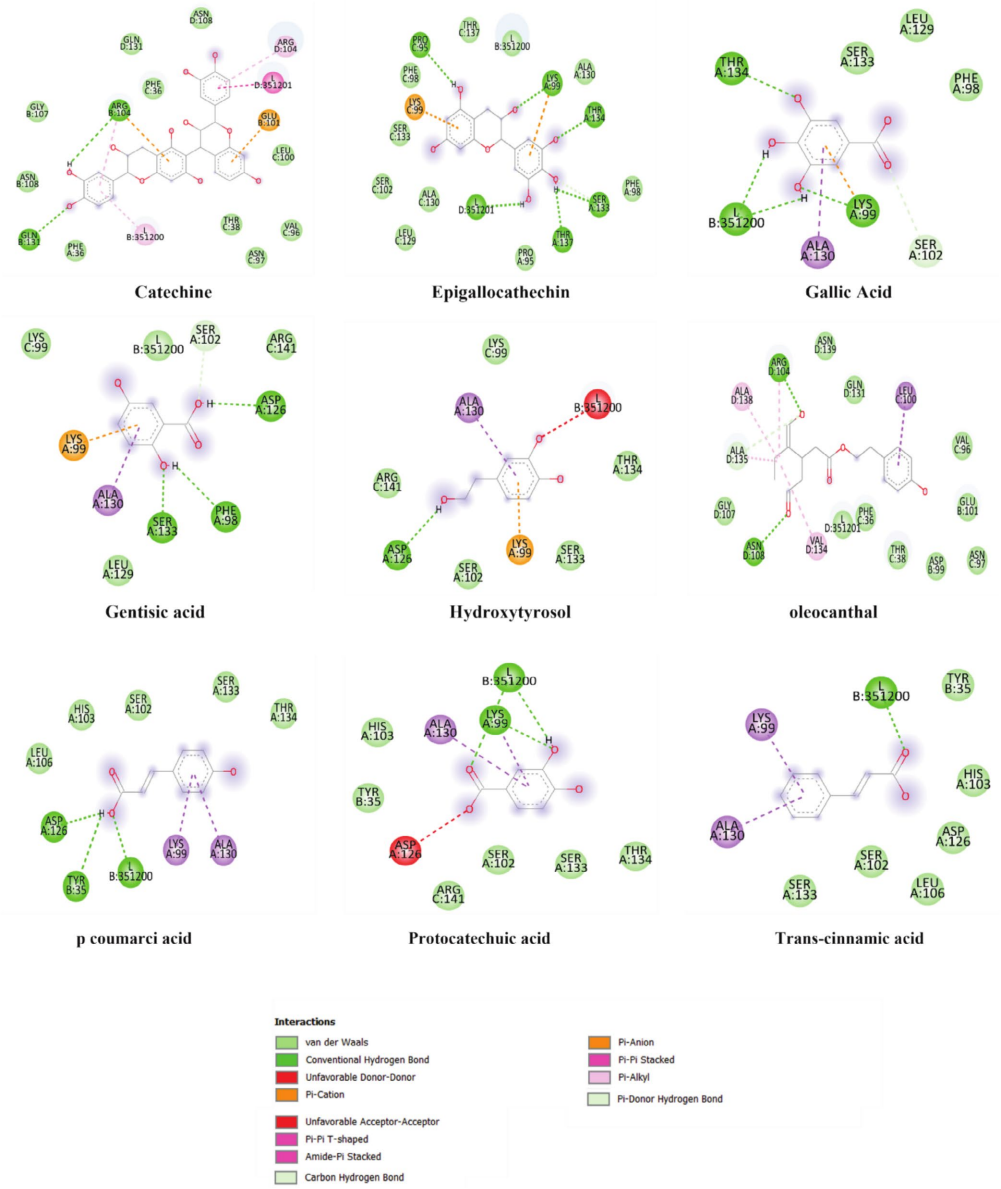
Ligands interactions with human hemoglobin protein (2D60).

Advanced glycation end products (AGEs), which are made up of reducing sugars and amino groups from proteins, have been linked to a number of chronic disorders in living things, including diabetes mellitus. Numerous studies have recently examined how phenolic compounds could prevent the production of AGEs (Peppa and Vlassara 2005). A phenolic acid called Gallic acid shows evidence of early‐stage glycation inhibition. Gallic acid's structural characteristics and antiglycation capacity are still unknown; however, they may be related to the antioxidant activity (Wu et al. 2010). In order to compare our compounds with Gallic acid's potential manner of binding to the active site residues of human hemoglobin protein (2D60), a molecular docking study was conducted. The human hemoglobin protein's active site and Gallic acid were linked through interactions like: THR134, L351200, ALA130, LYS99, SER102, PHE98, LEU129, SER133 (Figure 9), and a binding affinity of −5.8 kcal/mol. With the exception of hydroxytyrosol, all the ligands studied showed a great activity with the active site of human hemoglobin protein, especially catechin and epigallocatechin, with a binding affinities of −9.6 and −8.1 kcal/mol, respectively (Table 4), which confirms the in vitro results where some interesting findings of the extracts antiglycation activity were observed.

**TABLE 4:** The free binding energies (Kcal/mol) and interactions for the major compounds of *A. halimus* extracts against porcine pancreatic α‐amylase (1OSE) and human hemoglobin protein (2D60).

	1OSE	2D60
Catechin	−9,3	−9,6
Epigallocatechin	−8,3	−8,1
Gentisic acid	−5,9	−6,2
Hydroxytyrosol	−5,1	−5,3
Oleocanthal	−6,3	−6,5
Coumaric acid	−5,7	−6,4
Protocatechuic acid	−5,8	−5,9
Trans‐cinnamic acid	−5,6	−6,3
Acarbose	−8	
Gallic acid		−5,8

### 3.5 ADMET Prediction Analysis

According to the logS scale, substances have excellent solubility if their water solubility is between −4 and 0 (expressed in logS (log mol/l)) (Daina et al. 2017). Consequently, in this case, all the compounds are thought to be excellent water‐soluble chemicals. The Compounds 1, 2, 3, and 7 demonstrated poor Caco2 permeability, whereas the phytochemicals 4, 5, 6, and 8 had enhanced Caco2 permeability greater than 0.90 (reported as log P in 10^−6^ cm/s) and a high absorption rate in the human gastrointestinal tract. All phytochemicals are endowed with a skin permeability < −2.5, which refers to the fact that these compounds have high skin permeability. However, no substance has been revealed to be a P‐glycoprotein substrate, with the exception of catechin and epigallocatechin. According to absorption findings, none of the compounds were inhibitors of glycoproteins I or II (Table 4). Compounds 1, 2, 4, and 5 are well dispersed in plasma according to the steady‐state volume of distribution in humans, represented by VDss (provided in log L/Kg). Chemicals, with the exception of 1 and 2, are all considered to have reasonable blood–brain barrier permeability, 4, 5, 6, and 8 showed to have a reasonable penetration to the CNS, while 1, 2, 3, and 7 are unable to access the CNS (Table 5). The two major isoenzymes CYP2D6 and CYP3A4, which are crucial for drug metabolism, are used to categorize the metabolic interactions between compounds and cytochrome P450 (Pires et al. 2015). None of the previously listed compounds is a substrate or inhibitor of CYP2D6 or CYP3A4 (Table 4). Despite the fact that oleocanthal is not a substrate for renal OCT2 (organic cation transporter 2), all the compounds showed a high total clearance (mL/min/kg), with the highest being trans‐cinnamic acid at 0.781 log mL/min/kg (Table 5). None of the compounds in the table 4 appears to have an impact on how the human liver functions; Molecules 4 and 5 are the only ones to exhibit AMES toxicity. *hERG* gene inhibition by blocking potassium channels could cause ventricular arrhythmia (Sanguinetti and Tristani‐Firouzi 2006); however, none of the substances block this gene. None of the phytochemicals mentioned above could cause allergic contact dermatitis (Table 5).

**TABLE 5:** ADMET analysis of the major components identified in *Atriplex halimus* extracts via UHPLC–MS/MS: (1) catechin; (2) epigallocatechin; (3) gentisic acid; (4) hydroxytyrosol; (5) oleocanthal; (6) p‐coumaric acid; (7) protocatechuic acid; (8) trans‐cinnamic acid.

Component No.		1	2	3	4	5	6	7	8
Absorption	Water solubility	−3.11	−2.969	−2.00	−1.139	−2.703	−2.378	−2.069	−2.608
	Caco2 permeability	−0.28	−0.375	0.542	1.099	1.275	1.21	0.49	1.171
	Intestinal absorption (human) (%)	68.82	54.12	80.07	72.80	96.42	93.49	71.17	94.83
	Skin permeability	−2.73	−2.735	−2.73	−2.893	−2.792	−2.715	−2.727	−2.695
	P‐glycoprotein substrate	+	+	−	−	−	−	−	−
	P‐glycoprotein I inhibitor	−	−	−	−	−	−	−	−
	P‐glycoprotein II inhibitor	−	−	−	−	−	−	−	−
Distribution	VDss (human) (log L/kg)	1.027	1.301	−1.15	−0.084	0.068	−1.151	−1.298	−1.051
	Permeability BBB (log BB)	−1.054	−1.377	−0.69	−0.39	−0.447	−0.225	−0.683	0.446
	Permeability CNS (log PS)	−3.298	−3.507	−3.28	−2.672	−2.917	−2.418	−3.305	−1.834
Metabolism	CYP2D6 Sub	−	−	−	−	−	−	−	−
	CYP3A4 Sub	−	−	−	−	+	−	−	−
	CYP2D6 Inh	−	−	−	−	−	−	−	−
	CYP3A4 Inh	−	−	−	−	−	−	−	−
Excretion	Total clearance (log ml/min/kg)	0.183	0.328	0.587	0.23	0.555	0.662	0.551	0.781
	Renal OCT2 substrate	−	−	−	−	+	−	−	−
Toxicity	AMES toxicity	−	−	−	+	+	−	−	−
	Hepatotoxicity	−	−	−	−	−	−	−	−
	hERG I inhibitor	−	−	−	−	−	−	−	−
	Skin sensitivity	−	−	−	−	−	−	−	−

## 4 Conclusion

Our research demonstrated that *Atriplex halimus* extracts are notably rich in diverse bioactive compounds, as confirmed by UHPLC–MS/MS analysis. Furthermore, the various extract preparations exhibited significant inhibitory activity against two key diabetes‐related targets: pancreatic α‐amylase and hemoglobin glycation. Molecular docking studies performed on human pancreatic α‐amylase and human hemoglobin suggested plausible binding mechanisms for the major compounds identified in the extracts. Among these, catechin and epigallocatechin emerged as promising lead molecules for type 2 diabetes management, showing strong interactions with both target proteins. However, it is important to note that these findings are based on in vitro assays and in silico predictions, which may not fully replicate the complexity of biological systems in vivo. The study also focused on a limited number of targets, without exploring other relevant pathways involved in diabetes pathophysiology. Additionally, potential interactions between phytochemicals within the extracts, as well as their bioavailability and pharmacokinetic profiles, remain unaddressed. Therefore, further comprehensive studies, including in vivo models and clinical investigations, are essential to fully validate the antidiabetic potential of these phytochemicals.

## Ethics Statement

We confirm that prior to collecting *Atriplex halimus* (L.) for this study, explicit permission was obtained from the farm owner.

## Consent

The authors have nothing to report.

## Conflicts of Interest

The authors declare no conflicts of interest.

## Data Availability

Data involved in the present work are available from the corresponding author upon request.
